# Crystal Structure of the Kinase Domain of MerTK in Complex with AZD7762 Provides Clues for Structure-Based Drug Development

**DOI:** 10.3390/ijms21217878

**Published:** 2020-10-23

**Authors:** Tae Hyun Park, Seung-Hyun Bae, Seoung Min Bong, Seong Eon Ryu, Hyonchol Jang, Byung Il Lee

**Affiliations:** 1 Research Institute, National Cancer Center, Goyang, 10408 Gyeonggi, Korea; 74977@ncc.re.kr (T.H.P.); 97119@ncc.re.kr (S.-H.B.); 73138@ncc.re.kr (S.M.B.); hjang@ncc.re.kr (H.J.); 2 Department of Bioengineering, Hanyang University, 04763 Seoul, Korea; ryuse@hanyang.ac.kr; 3 Department of Cancer Biomedical Science, National Cancer Center Graduate School of Cancer Science and Policy, Goyang, 10408 Gyeonggi, Korea

**Keywords:** MerTK, AZD7762, TAM family kinase, X-ray crystallography

## Abstract

Aberrant tyrosine-protein kinase Mer (MerTK) expression triggers prosurvival signaling and contributes to cell survival, invasive motility, and chemoresistance in many kinds of cancers. In addition, recent reports suggested that MerTK could be a primary target for abnormal platelet aggregation. Consequently, MerTK inhibitors may promote cancer cell death, sensitize cells to chemotherapy, and act as new antiplatelet agents. We screened an inhouse chemical library to discover novel small-molecule MerTK inhibitors, and identified AZD7762, which is known as a checkpoint-kinase (Chk) inhibitor. The inhibition of MerTK by AZD7762 was validated using an in vitro homogeneous time-resolved fluorescence (HTRF) assay and through monitoring the decrease in phosphorylated MerTK in two lung cancer cell lines. We also determined the crystal structure of the MerTK:AZD7762 complex and revealed the binding mode of AZD7762 to MerTK. Structural information from the MerTK:AZD7762 complex and its comparison with other MerTK:inhibitor structures gave us new insights for optimizing the development of inhibitors targeting MerTK.

## 1. Introduction

Tyrosine-protein kinase Mer (MerTK) belongs to the family of Tyro3/Axl/Mer (TAM) receptor kinases, which are homeostatic regulators of reproduction, immune function, blood vessels, hematopoiesis, and the nervous system, mainly through the phagocytosis of apoptotic cells [1]. Proteins in the TAM family possess extracellular, transmembrane, and conserved kinase domains [2]. Although TAM family proteins share the common biological ligand growth-arrest-specific 6 (Gas6) for their functional activation [3], MerTK can also recognize other ligands, such as protein S and galectin-3, through its extracellular immunoglobulin and fibronectin-like domains [2,4,5]. The binding of these ligands to MerTK induces the autophosphorylation of tyrosine residues in the activation loop of the MerTK kinase domain (Tyr749, Tyr753, and Tyr754) [6], resulting in the recruitment of adaptor proteins including Grb2, LimD4, and Vav1 [7,8,9,10], and the activation of downstream enzymes such as PI3Ks, MAPKs, and GTPases [2,7].

Aberrant expression of MerTK was reported to promote the cell survival, invasive motility, and chemoresistance of a number of human cancers [5,11]. For example, MerTK is abnormally expressed in B and T cell acute lymphoblastic leukemia (ALL), but not expressed in normal mouse and human T and B lymphocytes in any stage of development [12,13]. The inhibition of MerTK by si/sh-RNA knockdown made cells more sensitive to chemotherapy and elevated the survival rate two-fold in a xenograft model of ALL. Similar results were also shown in non-small cell lung cancer (NSCLC) cells [14].

MerTK has also been reported to be a key factor in modulating macrophage activity and platelet aggregation [15]. MerTK activity promotes macrophage engulfment of materials released by apoptosis (a process called efferocytosis) and contributes to an anti-inflammatory response, suppressing the antitumor innate immune response within the tumor microenvironment [1,13,16,17]. MerTK knockout mice had decreased platelet aggregation without abnormal bleeding or significant changes in coagulation parameters [18]. This indicates that the inhibition of MerTK protects these mice from thrombosis without undesirable side effects, such as increased spontaneous bleeding.

Therefore, these observations indicate that MerTK is a potential drug target for human cancers and abnormal platelet aggregation. Research has also suggested that MerTK could be a new therapeutic target stimulating antitumor immune responses. To identify novel small molecules inhibiting MerTK activity, we carried out thermal shift assay-based drug screening with an inhouse kinase-inhibitor library and discovered a small molecule, AZD7762, which had been previously identified as a checkpoint-kinase (Chk) inhibitor [19,20]. Three clinical trials of AZD7762 have been conducted (ClinicalTrials.gov identifiers: NCT00937664, NCT00473616, and NCT00413686); however, these clinical trials were terminated due to unexpected cardiac toxicity [21]. We confirmed the specific binding of AZD7762 to the MerTK kinase domain by determining the three-dimensional structure of the MerTK kinase domain:AZD7762 complex. Structural information from the MerTK:AZD7762 complex gives us new insights into the development of MerTK inhibitors with high affinity and selectivity.

## 2. Results and Discussion

### 2.1. Screening for MerTK Binding Molecules with Inhouse Kinase-Inhibitor Library Using Thermal-Shift Assay

**Figure 1:**
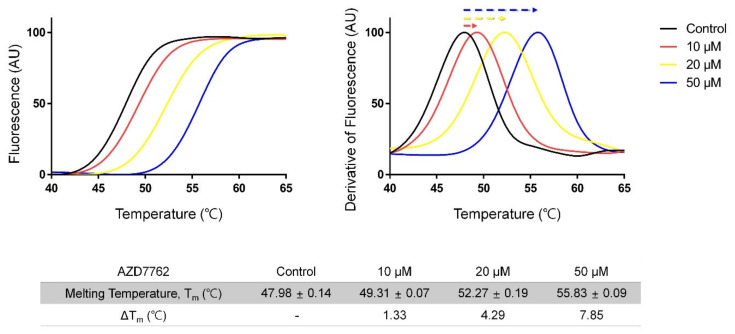
Thermal shift assay monitoring interaction between MerTK and AZD7762. (**left**) Melting curves; (**right**) corresponding derivative curves. The T_m_ of MerTK in the presence of 2% dimethyl sulfoxide (DMSO; negative control) was 47.9 °C. Addition of AZD7762 shifted T_m_ by 1.3, 4.3, and 7.9 °C, respectively. AU = arbitrary unit. Data are presented as mean ± standard deviation (SD) values of quadruplicate experiments.

We first screened for MerTK inhibitors with an inhouse kinase-inhibitor library (356 compounds) using a thermal shift assay, and found the eight hit compounds that showed a melting temperature (T_m_) shift of more than 1 °C (Table S1). Of these, it was suggested that AZD7762 (IUPAC name; (S)-5-(3-fluorophenyl)-N-(piperidin-3-yl)-3-ureidothiophene-2-carboxamide) has been shown to suppress metastatic spread and induce caspase 3/7-mediated apoptosis in breast-cancer cells by inhibiting Axl kinase signaling [22]. When we compared the T_m_ shift induced by AZD7762 for MerTK and Axl kinase domains, AZD7762 efficiently stabilized MerTK more than it did Axl, suggesting a stronger interaction with MerTK than with Axl (Figure 1 and Figure S1). These results are consistent with previous findings that AZD7762 has higher inhibitory efficacy toward MerTK than Axl kinase [22].

### 2.2. Verifying AZD7762 Inhibitory Activity by MerTK via HTRF Assay

**Figure 2:**
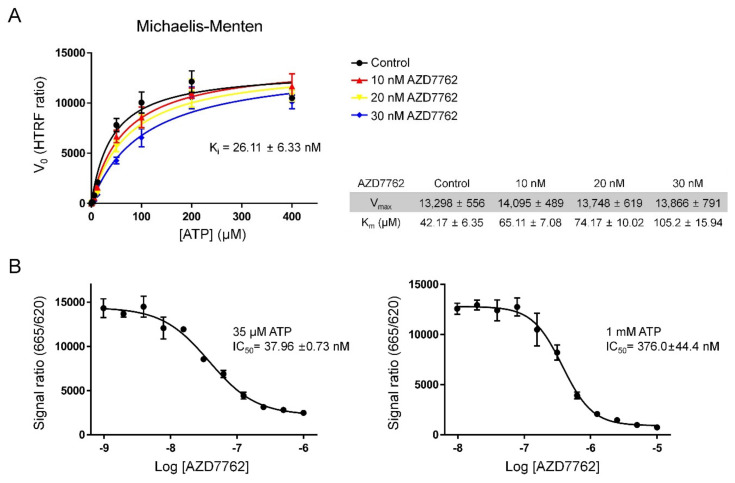
Homogeneous time-resolved fluorescence (HTRF) kinase assay. (**A**) Michaelis–Menten curve for MerTK kinase activity at different AZD7762 concentrations. (**B**) IC_50_ values of AZD7762 against MerTK at two different ATP concentrations. All measurements were fit to curves with the Michaelis–Menten or competitive-inhibition model using Graphpad Prism 6 (GraphPad Software, USA). All data presented as mean ± SD of quadruplicate experiments.

To test if AZD7762 could inhibit the kinase activity of MerTK, we performed a homogeneous time-resolved fluorescence (HTRF) assay (Figure 2). The Michaelis–Menten constant (K_m_) value for ATP, calculated by fitting the HTRF assay data into a Michaelis–Menten curve, was 42.17 (±6.35) µM, which is very similar to previously reported data (~40 µM) [23]. Experiments at different AZD7762 concentrations gave consistent maximal reaction rates (V_max_) values and different K_m_ values for ATP, which suggested that AZD7762 is a pure competitive inhibitor (Figure 2A). The calculated inhibitor constant (K_i_) obtained by fitting the data to a competitive inhibition model was 26.11 (±6.33) nM, and this K_i_ value was not significantly different from that reported in a previous study [22].

At 35 µM ATP (close to K_m_), the IC_50_ value of AZD7762 for MerTK kinase activity was ~37.96 (±0.73) nM. By setting the specific level of ATP to ~1 mM, which is similar to the intracellular concentration of ATP, the inhibitory activity of an inhibitor under physiological conditions can be approximated. Measurements of the IC_50_ at this high concentration of ATP are thus important to understand how in vitro activity may translate to in cellulo and ultimately in vivo inhibition. An IC_50_ value of 376.0 (±0.44) nM was obtained at 1 mM of ATP concentration (Figure 2B). Compared with the K_i_ value of AZD7762 for its primary target, Chk1 (3.6 nM), AZD7762 inhibited MerTK to a lesser extent [19]. However, AZD7762 could still provide a starting point for the development of effective MerTK inhibitors.

### 2.3. MerTK Inhibition by AZD7762 in Cells

**Figure 3:**
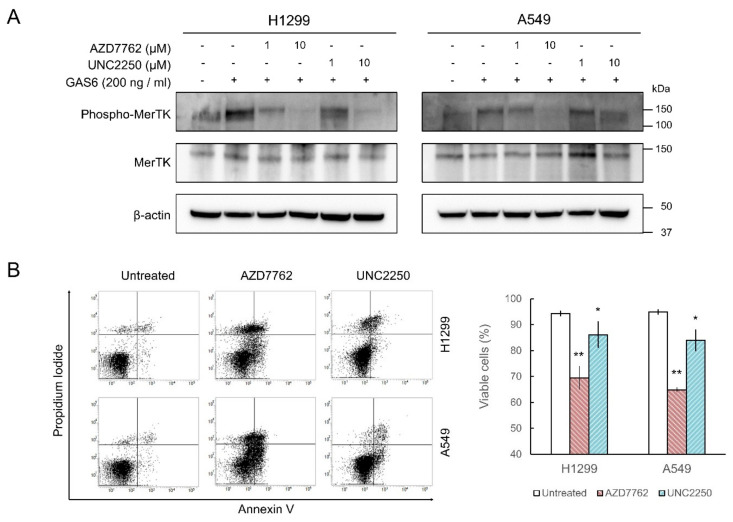
AZD7762 inhibits MerTK activity in lung cancer cell lines. (**A**) H1299 and A549 cells treated with AZD7762 or UNC2250 (positive control) for 4 h with indicated concentrations. After activation of MerTK by human Gas6, phosphorylated and total MerTK were detected by Western blot. β-actin was used as loading control. (**B**) Cells treated with 10 µM AZD7762 or UNC2250 for 48 h and stained with Annexin V (BD biosciences). Percentages of viable cells analyzed by flow cytometry and indicated as mean ± SD (*n* = 3). ** *p* < 0.001, * *p* < 0.05 relative to untreated cells.

To investigate the inhibitory activity of AZD7762 in cells, we treated non-small cell lung carcinoma cell lines (H1299 and A549 cells) with AZD7762. We chose these cell lines because their Gas6-induced endogenous TAM-dependent signaling activation is intact [24,25]. The increase in MerTK autophosphorylation after Gas6 treatment was monitored by Western blot. AZD7762 partially inhibited the increase in MerTK phosphorylation at 1 µM (~3 × IC_50_ at 1 mM ATP) and almost completely abolished phosphorylation at 10 µM in both cell lines (Figure 3A and Figure S2). UNC2250 [26], used as a positive control, also reduced MerTK phosphorylation; however, it was less effective than AZD7762 (Figure 3A). As knockdown of MerTK by small-hairpin RNA caused apoptosis in A549 cells [14], we investigated whether AZD7762 treatment induced apoptosis in lung cancer cells. Flow cytometric analysis after Annexin V and propidium iodide staining showed that AZD7762 treatment significantly induced apoptosis in both H1299 and A549 cells (Figure 3B). UNC2250 also caused apoptosis, but to a lesser extent (Figure 3B). These results suggest that AZD7762 inhibits MerTK in cells.

### 2.4. Crystal Structure of MerTK:AZD7762 Complex

To investigate how AZD7762 binds to the MerTK kinase domain, we determined the crystal structure of MerTK kinase domain in complex with AZD7762 at a resolution of 2.7 Å using X-ray diffraction data collected from co-crystals of the complex.

#### 2.4.1. Structure Determination and Model Quality

**Figure 4:**
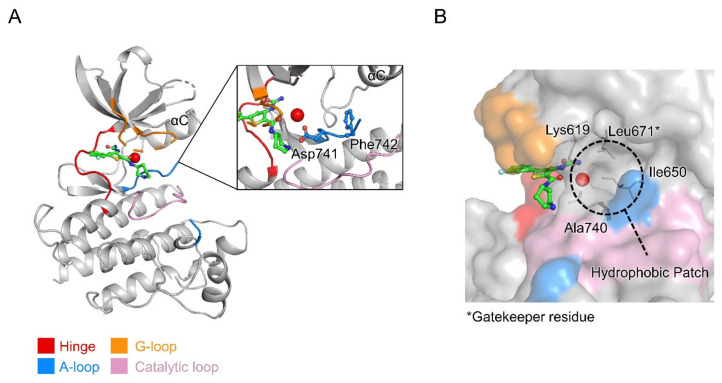
Overall crystal structure of MerTK kinase domain:AZD7762 complex. (**A**) Ribbon diagram for MerTK structure. Inset shows close up of ligand binding site with ordered water molecule, and Asp741 and Phe742 from the DFG motif at the N-terminus of the activation loop. (**B**) Surface representation of the ATP-binding site. Hydrophobic residues forming the hydrophobic patch, including Leu671, the gatekeeper residue of MerTK are drawn as sticks and labeled. Hinge region, glycine-rich loop, activation loop, and catalytic loop shown in red, orange, blue, and pink, respectively. Ordered water molecule near the hydrophobic patch represented with a red ball. Carbon atoms in AZD7762 molecule shown in green.

We determined the crystal structure of AZD7762-bound MerTK kinase domain (residues 571–864) by molecular replacement. The structural model was refined to R_work_ and R_free_ factors of 0.197 and 0.259, respectively. The crystal asymmetric unit contained two protein molecules (492 amino acid residues), 20 water molecules, and two AZD7762 molecules. There was no electron density for ~50 residues due to their flexibility; certain residues in the activation loop (A-loop; residues 744–762) and the glycine-rich loop (G-loop or P-loop; residues 594–598) were therefore missing from the model (Figure 4A). Refinement statistics are summarized in Table 1.

#### 2.4.2. Overall Structure of MerTK Kinase Domain:AZD7762 Complex

The structure of the MerTK kinase domain showed a typical protein kinase fold comprising two lobes, the N lobe (residues 578–670) and C lobe (residues 680–861), linked by a hinge region (residues 672–679) and the gatekeeper residue (Leu671) (Figure 4A). The ATP-binding pocket is located between the N and C lobes, enclosed by the hinge region and G-loop (residues 591–601).

Like other previously reported MerTK kinase domain structures, the MerTK:AZD7762 complex structure appears to be a partially inactive state. The C helix (αC) adopts a swung-out conformation from the ATP-binding pocket. In the swung-in conformation of αC characteristic of the active form of protein kinases, a glutamate residue in αC interacts with a lysine residue in the N lobe. In the MerTK:AZD7762 complex structure, Glu637 in αC does not interact with Lys619, resulting in the swung-out conformation of αC. However, the Asp–Phe–Gly (DFG) motif of the structure showed a DFG-in conformation, which is one of the structural features of active kinases. The side chain of Asp741 of the DFG motif is directed into the ATP-binding site, while that of Phe742 points toward the hydrophobic patch adjacent to the C helix (Figure 4A). The MerTK:AZD7762 structure suggests that AZD7762 may target the inactive form of MerTK, locking it in an inactive state, and can be categorized as a Type I 1/2 kinase inhibitor [27].

The hydrophobic residues near the ATP-binding site form a gate-like hydrophobic patch, which is one of the structural features of the TAM family kinase domain (Figure 4B) [28].

#### 2.4.3. Structural Analysis of MerTK:AZD7762 Complex and Comparison with AMPPNP-and Other Inhibitor-Bound Structures

**Figure 5:**
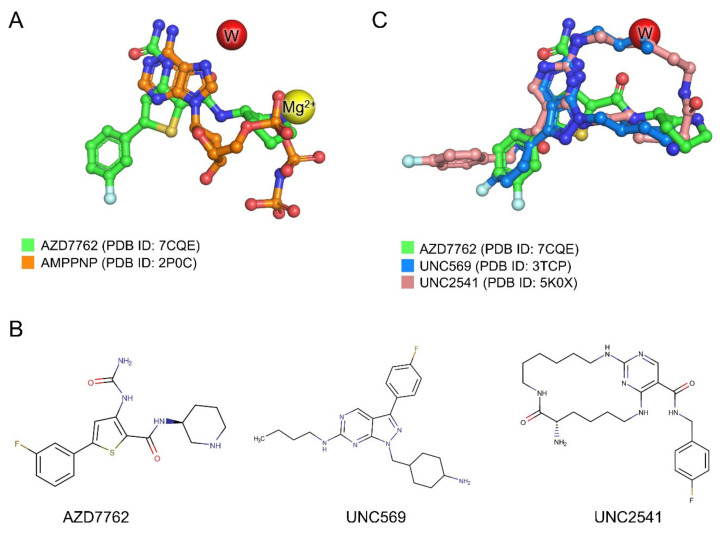
Structural superposition of ligand molecules in MerTK structures. (**A**) Structural superposition of AZD7762 and AMPPNP (ATP analog). (**B**) Chemical structures of AZD7762, UNC569, and UNC2541. (**C**) Structural superposition of AZD7762, UNC569, and UNC2541. The oxygen and nitrogen atoms in the chemical structures are shown in red and blue, respectively.

The AZD7762 molecule occupies the ATP-binding site of MerTK (Figure 4 and Figure 5A). The ureidothiophene carboxamide moiety of AZD7762 overlays with the adenine group of AMPPNP (an ATP analog), and its piperidine moiety is partially superimposed with the α phosphate group of AMPPNP (Figure 5A). We also compared the MerTK:AZD7762 complex structure with two representative MerTK:inhibitor complex structures, UNC569 (1-[(trans-4-aminocyclohexyl)methyl]-N-butyl-3-(4-fluorophenyl)-1h-pyrazolo[3,4-D]pyrimidin-6-amine) and UNC2541 ((7S)-7-amino-N-[(4-fluorophenyl)methyl]-8-oxo-2,9,16,18,21-pentazabicyclo[15.3.1]henicosa-1(21),17,19-triene-20-carboxamide), which are in pre-clinical studies for the treatment of various cancers [28,29,30,31].

These three inhibitors are structurally distinct (Figure 5B). AZD7762, UNC569, and UNC2541 have thiophene, pyrazolo[3,4-D] pyrimidine, or pyrimidine rings, respectively, as the hinge binding core. The core scaffolds of these three inhibitors overlay well in the adenine binding site of MerTK (Figure 5C). The fluorophenyl groups, the common moieties of the three inhibitors, show conformational diversity resulting in slightly different binding modes. As the fluorophenyl groups occupy the solvent-exposed region, it is likely to be difficult to increase affinity by changing the chemical structure of this moiety; however, changes to this moiety could be useful to improve the physicochemical properties of the inhibitors.

**Figure 6:**
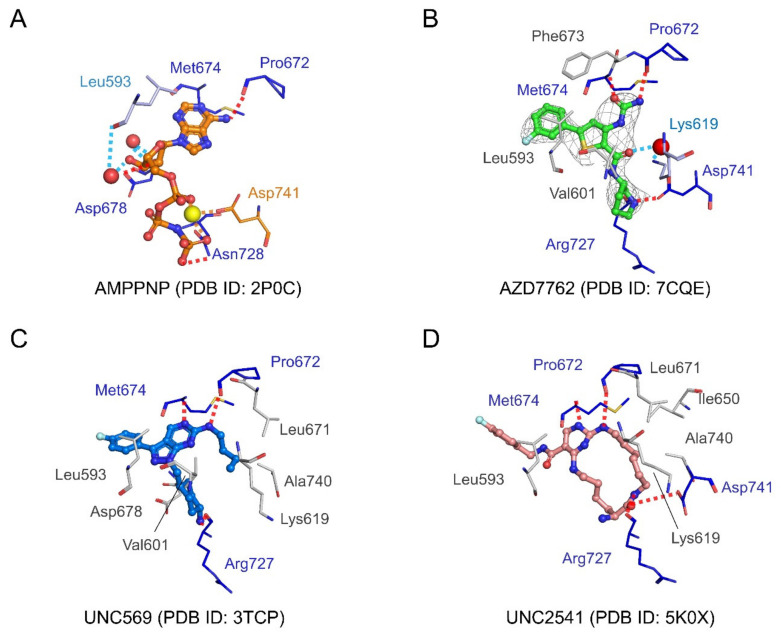
Detailed views of interactions between MerTK kinase domain and ligands (AMPPMP and inhibitors). (**A**–**D**) Detailed view of interactions between MerTK residues and AMPPNP, AZD7762, UNC569, and UNC2541. Residues involved in direct hydrogen bonds, water-mediated hydrogen bonds, and metal-mediated interactions highlighted in blue, light blue, and orange, respectively. Residues involved in hydrophobic interactions presented in gray. Hydrogen bonds and water-mediated interactions drawn as dashed red or sky-blue lines, respectively. Omit map for AZD7762 contoured at 1.0 σ.

All three compounds form three hydrogen bonds with Pro672 and Met674 from the hinge region, and Arg727 from the catalytic loop. Pro672 and Met674 also form hydrogen bonds with AMPPNP (Figure 6). AMPPNP makes additional hydrogen bonds with Asp678 and Asn728, and water-mediated hydrogen bonds with Leu593 and Asp678. A magnesium ion-mediated interaction network between Asp741 and the phosphate groups of AMPPNP was also found in the AMPPNP complex structure. Asp741 also makes hydrogen bonds with AZD7762 and UNC2541.

AZD7762 makes fewer hydrophobic interactions with MerTK than the UNC compounds. UNC569 and UNC2541 make extensive interactions with a hydrophobic patch formed of residues adjacent to the ATP-binding cleft, including Lys 619, Ile650, Leu671, and Ala740. These interactions are not present in the AZD7762 complex structure (Figure 4B and Figure 6). Hydrophobic interactions with Leu593 were found in all three inhibitor complex structures. Hydrophobic contacts with Val601 were found in AZD7762- and UNC569-bound MerTK structures, and with Phe673 in the AZD7762-bound structure. Asp678, which makes a hydrogen bond in the AMPPNP-bound structure, forms hydrophobic interactions with UNC569. In contrast to AMPPNP and AZD7762, UNC569 and UNC2541 make hydrophobic contacts with Leu671, the gatekeeper residue (Figure 6).

In the AZD7762 complex structure, a water-mediated interaction between the side-chain amino group of Lys619 and the carbonyl group in the carboxamide of AZD7762 was found. This bridges the ligand and the hydrophobic patch of MerTK (Figure 6). In the UNC569 and UNC2541 complex structures, the hydrophobic moieties of the inhibitors occupy the corresponding water position (Figure 5C). As mentioned above, Lys619 contributes to the αC helix conformation by interacting with Glu637, suggesting a structural basis for the partially inactive conformation of the MerTK:AZD7762 complex.

One route to develop AZD7762-derived MerTK inhibitors with high affinity could be to extend the AZD7762 molecule to interact with the residues of the hydrophobic patch. To this end, the carboxamide of AZD7762 is worth modification by introducing functional chemical groups that can generate hydrogen bonds or ion pairs with Lys619 to achieve higher affinity.

#### 2.4.4. Achieving Selectivity: Comparison of MerTK:AZD7762 Structure with Other TAM Family Kinases

**Figure 7:**
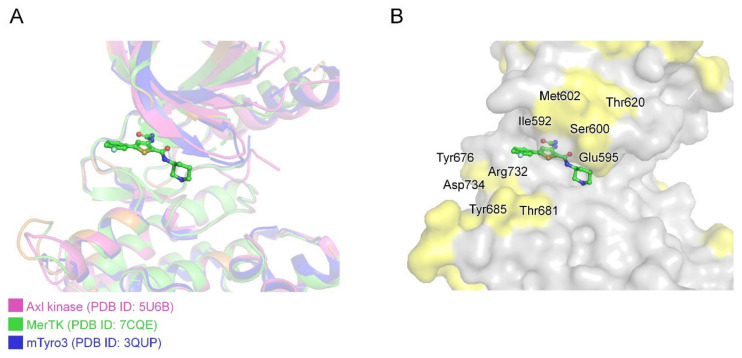
Structural alignment between TAM family kinase domain structures. (**A**) Superposition of Axl kinase, MerTK, and mTyro3 kinase domain structures presented in pink, green, and blue, respectively. (**B**) Surface representation of MerTK kinase domain structure; non-conserved residues in TAM family highlighted in yellow.

In further studies, to develop more effective anti-MerTK agents, the selectivity problem must be overcome. This problem is not only between kinases, but also between TAM family kinases. It is quite difficult to achieve selectivity between TAM family members because they are structurally very similar (Figure 7). In particular, gatekeeper residues are identical among TAM family kinases. Published MerTK inhibitors such as UNC 569, UNC1062, UNC1666, and UNC2025 appear to have some selective inhibitory activity within the TAM family; however, their IC_50_ values for MerTK were only ~10 times better than those for Axl and Tyro3 [30,32,33,34]. In other words, they can still efficiently inhibit Axl and Tyro3. Structural comparison of TAM kinases (human MerTK, human Axl, and mouse Tyro3) alongside amino acid sequence alignment (Figure 7 and Figure S3) showed that the hydrophobic-patch region is strictly conserved and cannot be utilized for selective inhibitor development. However, residues around the G-loop (Ile592, Glu595, Ser600, and Met602) and C-lobe (Tyr676, Thr681 Tyr685, Arg732, and Asp734) are less conserved in TAM kinases (Figure 7B), and a selective MerTK inhibitor could be developed by chemical elaboration toward these non-conserved regions. Introduction of various chemical groups into the solvent-exposed fluorophenyl group of AZD7762 could provide a route to increase selectivity towards MerTK, and this could improve the physicochemical properties, as mentioned above, at the same time.

A series of studies revealed that double or triple knockout in the TAM family caused more intense immune responses, including a hyperinflammatory state with severe autoimmunity, multiple organ defects, massive lymphoproliferation, and neoplasia, such as colitis-associated colon cancer [35,36,37,38,39]. The predicted side effects of triple downregulation by nonselective inhibitors therefore appear to be one of the main obstacles that should be overcome in the development of effective inhibitors targeting MerTK. Hurdles must be overcome to improve the druggability of anti-MerTK inhibitors, including the selectivity problem mentioned above. So far, one series of MerTK-selective inhibitors has been developed; however, their selectivity among the three TAM family kinases remains insufficient [35] and more highly selective MerTK inhibitors still need to be developed. The structural insights from the MerTK:AZD7762 structure described herein will provide novel strategies to develop such inhibitors.

### 2.5. Concluding Remarks

In summary, we verified the inhibitory activity of AZD7762 for MerTK using in vitro kinase and cell-based assays. In addition, direct binding to MerTK was proven by determining the crystal structure of the MerTK kinase domain:AZD7762 complex. By comparing with other MerTK inhibitor-bound structures, we identified the unique features of the binding mode of AZD7762 and gained structural insights to guide the development of more effective MerTK inhibitors. As the primary targets of AZD7762 are Chk kinases and MerTK is one of AZD7762’s promiscuous targets, extensive structure-guided derivative studies for AZD7762 are required for development of highly MerTK-specific inhibitors with high efficacy and fewer side effects.

## 3. Materials and Methods

### 3.1. Protein Expression and Purification

The MerTK kinase domain (571–864) and protein-tyrosine phosphatase 1B (PTPN1) were subcloned into MCS1 and MCS2 of pETDuet1 (Merck Millipore, Burlington, MA, USA), respectively. In the case of MerTK, the cDNA sequence of the kinase domain (571–864) was subcloned between the NcoI and NotI restriction enzyme sites. Resulting recombinant MerTK had a fusion tag that contained the TEV (Tobacco Etch Virus) protease cleavable polyhistidine (6 × His) tag at its N terminus.

The recombinant proteins were expressed in *Escherichia coli* BL21 (DE3) cells (Merck Millipore, Burlington, MA, USA). The cells were initially cultured in Terrific Broth at 37 °C. Proteins were induced with 0.1 mM isopropyl-β-D-thiogalactoside (IPTG) when the cells had reached an optical density (OD_600_) of 0.6 and the cells were further cultured for approximately 16 h at 15 °C. The cells were harvested by centrifugation at 11,000× *g* for 15 min at 4 °C.

The cells were resuspended and lysed in lysis buffer (50 mM Tris-HCl pH 8.0, 500 mM NaCl, 1 mM β-mercaptoethanol, 1 mM phenylmethylsulfonyl fluoride) by passing through a microfluidizer (PICOMAX, Micronox, Sungnam, Korea) three times with 1000 bar pressure. After centrifugation at 13,000× *g* for 1 h, the supernatant was incubated with Ni-NTA resin (GE healthcare, Chicago, IL, USA) with gentle stirring for 1 h under ice-cold conditions. The Ni-NTA resin was washed and the bound proteins were eluted with imidazole containing buffers (50 mM Tris-HCl pH 8.0, 500 mM NaCl, 2 mM β-mercaptoethanol, 30, 40, 50, 70, and 300 mM imidazole) stepwise. After the MerTK-containing fractions (40, 50, and 70 mM imidazole) were pooled, the protein was further purified using a HiLoad 16/600 Superdex 200 prep grade column (GE Healthcare, Chicago, IL, USA) equilibrated and run with final storage buffer (20 mM Tris-HCl pH 8.0, 500 mM NaCl, 2 mM β-mercaptoethanol). The protein was concentrated to 40 mg/mL using Amicon Centrifugal Filter Units (Merck Millipore, Burlington, MA, USA) and stored at −80 °C until use for crystallization.

### 3.2. Thermal-Shift Assay

First, we screened for MerTK inhibitors with an inhouse kinase library using the thermal shift assay method. To the mixed solutions (0.5 mg/mL MerTK and 10 µM library compounds in reaction buffer (20 mM Tris-HCl, pH 8.0, 0.5 M NaCl, 2 mM DTT)) was added protein thermal shift dye (from Protein Thermal Shift™ Dye Kit, 1× final concentration; Applied Biosystems, Waltham, MA, USA). To avoid finding too many hits with low affinity, we used compounds at low concentration. The shifts of the T_m_ were measured and visualized with Quantstudio 6 real time PCR (Applied Biosystems, Waltham, MA, USA).

AZD7762 (Sigma-Aldrich, St. Louis, MO, USA) was incubated with MerTK and Axl kinase (0.5 mg/mL) at three different concentrations (10 µM, 20 µM, and 50 µM) for the dose–response thermal shift assay.

### 3.3. HTRF Assay

The inhibitory activity of AZD7762 against MerTK was measured and verified with an HTRF assay, an application of the fluorescence resonance energy-transfer (FRET) technique. The HTRF KinEASE-TK kit (Cisbio, Bedford, MA, USA) was used according to the manufacturer’s instructions. To draw and fit the Michaelis–Menten curves, an HTRF assays at various concentrations of ATP were performed. IC_50_ values were obtained at 35 µM (close to K_m_ value for ATP from in vitro assay) or 1 mM ATP (ATP concentration in cells), respectively. We used 1 ng/µL of purified recombinant MerTK for the kinase assay, and the reaction mixture contained 0.2% DMSO.

Fluorescence signals were measured at two wavelengths for the HTRF assay. The excitation wavelength was 320 nm, and the emission wavelengths were 620 and 665 nm. Fluorescence was detected using an Infinite F200pro with a lag time of 150 µs and integration time of 500 µs (Tecan, Männedorf, Switzerland). Data reduction was carried out following the manufacturer’s guidelines (Ratio = 10,000 × signal 665 nm/signal 620 nm).

### 3.4. Crystallization and Structure Determination

Crystallization experiments were performed using the sitting-drop vapor-diffusion method at 14 °C. Before crystallization, the MerTK kinase domain protein stock was incubated with 5 mM AZD7762 (approximately 1:5 protein:compound molar ratio) at 4 °C overnight. Crystallization drops were set up by mixing 0.5 µL of the compound–protein solution and 0.5 µL of reservoir solution (100 mM Tris-HCl, pH 8.5, 4 M sodium chloride). Crystals grew within 5 days.

Diffraction data were collected using a Dectris Pilatus 6M CCD detector at the BL-11C experiment station of the Pohang Light Source (Pohang, Korea). Crystals were cryoprotected using cryobuffer (reservoir solution supplemented with 2.5 mM AZD7762 and 30% (*v*/*v*) glycerol). Diffraction data were processed and scaled using the HKL2000 program suite [40]. The structure was solved by molecular replacement with PHASER [41] using a MerTK structure (PBD entry, 4MH7) as the search model [42]. Model building was completed using COOT [43], and refinement was performed with phenix.refine in the Phenix program suite [44]. Coordinates and cif restraint files for AZD7762 were generated using Maestro version 2017-3 (Schrödinger, New York, NY, USA) and eLBOW in the Phenix program suite, respectively [45,46]. Data collection and refinement statistics are listed in Table 1. The structural figures were drawn using Pymol, ver. 2.3.0 (Schrödinger, New York, NY, USA).

### 3.5. Cell Culture

The H1299 and A549 cell lines were provided by the Korean Cell Line Bank (Seoul, Korea). Cells were cultured in an RPMI-1640 medium (Hyclone, Logan, UT, USA) supplemented with 10% fetal bovine serum (Hyclone, Logan, UT, USA), and 1% penicillin–streptomycin (Invitrogen, Carlsbad, CA, USA) at 37 °C with 5% CO_2_ in a humidified cell culture incubator. The cell line was authenticated and checked for *Mycoplasma* at the Genomics Core Facility (National Cancer Center, Korea) as described previously [47].

### 3.6. Chemical Treatment and Western Blotting

The H1299 and A549 cells were incubated in a serum-free medium for 4 h with AZD7762 (1 µM, 10 µM, and 20 µM). Prior to cell harvesting, 200 ng/mL recombinant human Gas6 (R&D Systems, Minneapolis, MN, USA) was added for 20 min to activate MerTK. As a positive control, equal concentrations of UNC22520 were used. Western blotting was performed according to a modified version of a previously described method [48]. Cells were harvested and washed in phosphate-buffered saline (PBS), and then lysed in a buffer containing 20 mM Tris-HCl (pH 7.4), 150 mM NaCl, 1% (*v*/*v*) Triton X-100, 1 mM EDTA, a protease inhibitor cocktail (P3100; genDEPOT, Katy, TX, USA), and a phosphatase inhibitor cocktail (P3200; genDEPOT, Katy, TX, USA). After SDS-PAGE and protein transfer, the desired protein was detected using an iBind™ Automated Western System (Thermo Fisher Scientific, Waltham, MA, USA). Anti-MerTK antibody (ab52968) was purchased from Abcam (Cambridge, UK), antiphospho-MerTK antibody (p186-749) was from PhosphoSolutions (Aurora, CO, USA), and anti-β-actin antibody (A2226) was from Sigma-Aldrich (St. Louis, MO, USA).

### 3.7. Flow-Cytometry Analysis for Apoptosis

H1299 and A549 cells treated with 10 µM AZD7762 or UNC2250 for 48 h were analyzed by annexin V and propidium iodide staining, and flow cytometry at the Flow Cytometry Core Facility (National Cancer Center) using FACSVerse (BD Bioscience, San Jose, CA, USA), as described previously [49].

### 3.8. Statistics

Statistical analyses were performed as described previously [50]. *P* values were calculated using GraphPad version 7.0d (GraphPad Software Inc., Cary, NC, USA), and those less than 0.05 were considered significant.

## Figures and Tables

**Table 1:** Data collection and refinement statistics.

Dataset	MerTK Kinase Domain: AZD7762
**Diffraction-data statistics**	
X-ray source	PLS-11C
Wavelength (Å)	0.979
Space group	C2
Cell parameters	
a, b, c (Å)	116.12, 69.20, 92.61
α, β, γ (°)	90.00, 118.42, 90.00
Resolution range (Å) ^a^	50.0−2.70 (2.75−2.70)
R_sym_ (%) ^b^	10.3 (40.0)
R_p.i.m._ (%) ^c^	5.1 (21.0)
CC_1/2_	0.986 (0.875)
Mean I/σI	13.8 (3.33)
Redundancy	4.7 (4.0)
Completeness (%)	95.9 (84.2)
No. of unique reflections	17,283 (738)
**Refinement statistics**	
Resolution range (Å)	43.53−2.70
R_work_/R_free_ (%) ^d^	19.7/25.9
No. of nonhydrogen atoms/average B-factor (Å^2^)	
Protein	3948/41.47
Solvent	20/22.65
Ligand (AZD7762)	56/37.25
R.m.s. deviation	
Bond lengths (Å)	0.015
Bond angles (°)	1.97
Ramachandran plot (%)	
Favored/outliers	97.46/0.42
Clash score	3.0
PDB entry	7CQE

^a^ Values in parentheses are for the highest-resolution shell. ^b^ R_sym_ = Σ_h_ Σ_i_ | I(h)_i_ – < I(h) > |/Σ_h_ Σ_i_ I(h)_i_, where I(h) is the intensity for reflection h, Σ_h_ is the sum for all reflections, and Σ_i_ is the sum for i measurements of reflection h. ^c^ R_p.i.m._ = Σ_h_ (1/(n-1))^1/2^ Σ_i_ | I(h)_i_ – < I(h) > |/Σ_h_ Σ_i_ I(h)_i_, where I(h) is the intensity for reflection h, Σ_h_ is the sum for all reflections, and Σ_i_ is the sum for i measurements of reflection h. ^d^ R = Σ | |F_obs_| – |F_calc_| |/Σ |F_obs_|, where R_free_ is calculated using a randomly chosen 10% of reflections that were not used for structure refinement, and R_work_ is calculated for the remaining reflections.

## Supplementary Materials

The following are available online at https://www.mdpi.com/1422-0067/21/21/7878/s1. Table S1: Thermal shift-based MerTK inhibitor screening results; Figure S1: Thermal shift assay monitoring the interaction between Axl kinase and AZD7762; Figure S2: Uncropped images of western blots presented in Figure 3; Figure S3: Amino acid sequence alignment of human Tyro3, Axl, and MerTK kinase domains.

## Abbreviations

Grb2	Growth factor receptor-bound protein 2
LimD4	Lim domain-containing protein 4
Vav1	Guanine nucleotide-exchange factor (GEF) Vav1
PI3K	Phosphoinositide 3-kinase
ATP	Adenosine triphosphate
AMPPNP	Adenylyl-imidodiphosphate
