# Supplementary material for: Crystal Structure of the Kinase Domain of MerTK in Complex with AZD7762 Provides Clues for Structure-Based Drug Development
Source: Int J Mol Sci. 2020 Oct 23;21(21):7878. doi: 10.3390/ijms21217878 (PMC7660649; doi:10.3390/ijms21217878)
Supplement: Supplementary file 1 [file ijms-21-07878-s001.pdf]

Supplementary Materials

Table S1. Thermal shift-based MerTK inhibitor screening results.

Number	Compound	ΔT_m (℃)[a]
#1	Linifanib (ABT-869)	0.16
#2	WZ8040	0.16
#3	CAL-101 (Idelalisib, GS-1101)	-0.19
#4	Torin 2	-0.09
#5	**Cediranib (AZD2171)**	1.54
#6	AG-1024	0.27
#7	Honokiol	0.16
#8	Golvatinib (E7050)	-0.04
#9	Imatinib Mesylate (STI571)	-0.04
#10	Zoledronic Acid	0.01
#11	PP121	-0.04
#12	Tyrphostin 9	0.37
#13	Rapamycin (Sirolimus)	-0.70
#14	Pelitinib (EKB-569)	0.62
#15	NU7441 (KU-57788)	-0.19
#16	Icotinib	0.21
#17	Enzastaurin (LY317615)	0.52
#18	GSK429286A	0.01
#19	Ibrutinib (PCI-32765)	0.11
#20	VX-702	0.21
#21	SB216763	0.01
#22	CCT129202	0.57
#23	GDC-0980 (RG7422)	0.83
#24	XL019	0.42
#25	KU-55933 (ATM Kinase Inhibitor)	0.32
#26	R406 (free base)	0.62
#27	PH-797804	-0.19
#28	AZD3463	0.88
#29	LY294002	0.62
#30	KU-60019	0.37
#31	PHA-767491	-0.04
#32	AVL-292	0.16
#33	JNJ-38877605	0.16
#34	PF-573228	0.32

#35	HER2-Inhibitor-1	0.42
#36	CNX-774	0.21
#37	AT9283	0.93
#38	BGJ398 (NVP-BGJ398)	0.73
#39	Dovitinib (TKI-258) Dilactic Acid	0.46
#40	AR-A014418	0.21
#41	Lenvatinib (E7080)	0.57
#42	OSI-420	0.57
#43	WP1066	0.16
#44	AZD5363	0.72
#45	Axitinib	0.01
#46	ENMD-2076	0.37
#47	PIK-294	-0.14
#48	TAE226 (NVP-TAE226)	0.01
#49	Dovitinib (TKI-258, CHIR-258)	0.57
#50	Amuvatinib (MP-470)	-0.04
#51	Indirubin	-0.14
#52	IMD 0354	0.52
#53	Lapatinib (GW-572016) Ditosylate	0.32
#54	Genistein	0.98
#55	OSI-027	0.37
#56	ZM 323881 HCl	0.32
#57	Sorafenib Tosylate	0.37
#58	Aurora A Inhibitor I	0.57
#59	GSK2126458 (GSK458)	0.32
#60	CHIR-99021 (CT99021) HCl	0.47
#61	AC480 (BMS-599626)	0.11
#62	Pimasertib (AS-703026)	0.37
#63	AS-604850	0.21
#64	AP26113	0.72
#65	SB203580	0.78
#66	SAR245409 (XL765)	0.62
#67	A-769662	0.37
#68	PD168393	0.52
#69	GSK1904529A	0.42
#70	CP-673451	0.11
#71	Dacomitinib (PF299804, PF299)	0.37
#72	NU6027	0.27

#73	OSU-03012 (AR-12)	0.72
#74	BS-181 HCl	0.83
#75	PF-04691502	-0.04
#76	SKI II	-0.04
#77	Palbociclib (PD-0332991) HCl	0.47
#78	BMS-265246	0.21
#79	Varlitinib	0.78
#80	CO-1686 (AVL-301)	0.16
#81	Brivanib Alaninate (BMS-582664)	0.57
#82	AST-1306	0.83
#83	MK-5108 (VX-689)	0.62
#84	GSK2636771	-0.04
#85	CP-724714	0.11
#86	PIK-293	0.01
#87	AZD4547	0.27
#88	TCS 359	0.06
#89	Saracatinib (AZD0530)	0.16
#90	CUDC-101	0.36
#91	Telatinib	-0.25
#92	Tideglusib	-0.19
#93	Canertinib (CI-1033)	0.57
#94	JNJ-7706621	0.42
#95	Quercetin	0.32
#96	WHI-P154	0.01
#97	Motesanib Diphosphate (AMG-706)	-0.25
#98	TG100-115	0.72
#99	Fostamatinib (R788)	0.06
#100	ZM 306416	0.11
#101	Sunitinib Malate	0.62
#102	PHA-680632	0.27
#103	WYE-125132 (WYE-132)	0.27
#104	TAK-715	-0.09
#105	Masitinib (AB1010)	0.72
#106	HMN-214	0.47
#107	CAY10505	0.32
#108	Unknown compound	0.32
#109	SB202190 (FHPI)	0.72
#110	AT7519	0.06

#111	CH5132799	0.42
#112	AZ20	0.57
#113	PF-04217903	0.47
#114	AZD8055	0.57
#115	AG-1478 (Tyrphostin AG-1478)	0.16
#116	TIC10 Analogue	-0.19
#117	Danusertib (PHA-739358)	0.27
#118	Fasudil (HA-1077) HCl	0.57
#119	CCT137690	-0.30
#120	GZD824	0.37
#121	Triciribine	0.52
#122	AZD8330	0.27
#123	Wortmannin	0.21
#124	TAK-632	0.01
#125	AG-490 (Tyrphostin B42)	0.27
#126	AZD8931 (Sapitinib)	0.83
#127	MK-2461	0.16
#128	PQ 401	0.67
#129	TGX-221	0.42
#130	AZ 960	0.11
#131	CEP-33779	0.37
#132	Tyrphostin AG 1296	0.57
#133	Selumetinib (AZD6244)	-0.09
#134	PIK-75 HCl	0.11
#135	Volasertib (BI 6727)	0.14
#136	TPCA-1	-0.19
#137	PD184352 (CI-1040)	0.32
#138	PD173074	0.67
#139	Chrysophanic Acid	0.11
#140	TG100713	-0.09
#141	Nilotinib (AMN-107)	0.01
#142	GSK1059615	0.21
#143	LY2603618	0.11
#144	GNF-2	-0.04
#145	Tandutinib (MLN518)	0.37
#146	VX-745	0.01
#147	A-674563	0.01
#148	Pazopanib	0.83

#149	GDC-0941	0.27
#150	AEE788 (NVP-AEE788)	0.21
#151	CHIR-124	0.21
#152	MEK162 (ARRY-162, ARRY-438162)	-0.30
#153	MK-2206 2HCl	-0.35
#154	Quizartinib (AC220)	0.21
#155	KX2-391	-0.70
#156	PP1	0.32
#157	MLN8054	0.27
#158	PHT-427	0.52
#159	SB415286	0.25
#160	CGK 733	0.06
#161	TAE684 (NVP-TAE684)	0.21
#162	BIRB 796 (Doramapimod)	0.21
#163	CHIR-98014	0.27
#164	RKI-1447	-0.60
#165	XL147 analogue	0.83
#166	Neratinib (HKI-272)	0.98
#167	CUDC-907	-0.19
#168	ZCL278	0.47
#169	SNS-032 (BMS-387032)	0.42
#170	GSK461364	0.21
#171	AZD2014	-0.09
#172	ZM 39923 HCl	0.27
#173	WZ3146	0.42
#174	Mubritinib (TAK 165)	0.42
#175	Dabrafenib (GSK2118436)	-0.14
#176	NSC 23766	0.32
#177	Dactolisib (BEZ235, NVP-BEZ235)	0.16
#178	Tivozanib (AV-951)	0.67
#179	Palomid 529 (P529)	-0.30
#180	Torin 1	0.11
#181	Dasatinib	0.06
#182	WYE-354	0.11
#183	Imatinib (STI571)	-0.04
#184	GW5074	0.52
#185	NVP-AEW541	0.37
#186	MGCD-265	0.72

#187	Gedatolisib (PF-05212384, PKI-587)	-0.09
#188	S-Ruxolitinib (INCB018424)	-0.04
#189	Temsirolimus (CCI-779, NSC 683864)	-0.09
#190	Thiazovivin	-0.04
#191	**AS-252424**	1.18
#192	Piceatannol	0.11
#193	SL-327	0.62
#194	PHA-793887	-0.19
#195	NVP-BSK805 2HCl	0.47
#196	PP2	-0.35
#197	SU11274	0.16
#198	Hesperadin	0.88
#199	GSK1838705A	-0.35
#200	MK-8745	0.62
#201	Vatalanib (PTK787) 2HCl	0.06
#202	KRN 633	0.42
#203	Crenolanib (CP-868596)	-0.04
#204	AZD1080	-0.09
#205	BI 2536	0.47
#206	Tie2 kinase inhibitor	0.42
#207	AZ 628	0.83
#208	BIO	0.11
#209	Cabozantinib (XL184, BMS-907351)	0.93
#210	KW-2449	0.54
#211	NVP-BVU972	0.37
#212	WZ4003	0.52
#213	Barasertib (AZD1152-HQPA)	0.21
#214	R406	0.88
#215	TAK-285	-0.19
#216	SMI-4a	0.42
#217	CYC116	0.52
#218	PP242	0.57
#219	GDC-0068	-0.09
#220	PRT062607 (P505-15, BIIB057) HCl	0.01
#221	Nintedanib (BIBF 1120)	0.01
#222	YM201636	-0.04
#223	Degrasyn (WP1130)	-0.35
#224	SAR131675	-0.19

#225	Ridaforolimus (Deforolimus, MK-8669)	-0.09
#226	Vemurafenib (PLX4032, RG7204)	0.16
#227	Phenformin HCl	-0.76
#228	IKK-16 (IKK Inhibitor VII)	-0.09
#229	Pazopanib HCl	0.21
#230	Rigosertib (ON-01910)	0.57
#231	DCC-2036 (Rebastinib)	0.01
#232	PF-477736	0.47
#233	Vandetanib (ZD6474)	0.32
#234	SP600125	0.11
#235	PF-00562271	0.16
#236	SC-514	-0.14
#237	**Crizotinib (PF-02341066)**	1.29
#238	PIK-93	0.57
#239	R547	-0.09
#240	CZC24832	-0.19
#241	Brivanib (BMS-540215)	-0.14
#242	BIX 02188	0.52
#243	TAK-901	-0.04
#244	LDK378	0.11
#245	U0126-EtOH	0.27
#246	AT7867	-0.50
#247	Aloin	0.16
#248	10058-F4	0.72
#249	Foretinib (GSK1363089)	-0.09
#250	H 89 2HCl	0.37
#251	AMG-458	0.78
#252	Bisindolylmaleimide IX (Ro 31-8220 Mesylate)	-0.25
#253	Everolimus (RAD001)	0.16
#254	**RAF265 (CHIR-265)**	1.03
#255	Alectinib (CH5424802)	0.11
#256	EHop-016	-0.60
#257	PLX-4720	0.01
#258	SGI-1776 free base	0.52
#259	Capmatinib (INCB28060)	-0.04
#260	VE-821	-0.45
#261	WZ4002	0.16
#262	CYT387	0.11

#263	INK 128 (MLN0128)	-0.04
#264	Butein	0.98
#265	Afatinib (BIBW2992)	0.32
#266	OSI-930	0.01
#267	BKM120 (NVP-BKM120, Buparlisib)	-0.19
#268	BI-D1870	-0.35
#269	Erlotinib HCl (OSI-744)	0.01
#270	BX-795	0.52
#271	TAK-733	0.01
#272	PF-562271	0.27
#273	PD0325901	-0.40
#274	Ki8751	-0.04
#275	CCT128930	-0.45
#276	Go 6983	-0.81
#277	VX-680 (Tozasertib, MK-0457)	0.11
#278	AZD6482	0.32
#279	Trametinib (GSK1120212)	0.01
#280	Tofacitinib (CP-690550) Citrate	-0.09
#281	PHA-665752	-0.25
#282	Ponatinib (AP24534)	0.27
#283	WAY-600	-0.40
#284	IPI-145 (INK1197)	0.01
#285	NVP-ADW742	-0.19
#286	BIX 02189	0.57
#287	AMG-900	-0.19
#288	IPA-3	0.42
#289	ZM 447439	0.37
#290	**BMS-777607**	1.44
#291	TG101348 (SAR302503)	0.21
#292	LY2835219	0.16
#293	SGX-523	0.27
#294	TWS119	0.52
#295	BGT226 (NVP-BGT226)	0.37
#296	Skepinone-L	-0.86
#297	BMS-754807	0.67
#298	PF-4708671	0.21
#299	3-Methyladenine	-0.09
#300	TG003	0.06

#301	Roscovitine (Seliciclib,CYC202)	0.06
#302	**BMS-794833**	1.34
#303	Tofacitinib (CP-690550,Tasocitinib)	0.07
#304	AG-18	0.01
#305	PD98059	0.67
#306	SB590885	0.01
#307	BYL719	0.27
#308	GDC-0349	0.32
#309	Bosutinib (SKI-606)	0.62
#310	KU-0063794	-0.04
#311	Asiatic Acid	0.16
#312	Semaxanib (SU5416)	0.42
#313	Gefitinib (ZD1839)	0.01
#314	BX-912	-0.09
#315	AZD5438	-0.14
#316	NU7026	-0.19
#317	PI-103	-0.70
#318	Ruxolitinib (INCB018424)	-0.70
#319	A66	-0.60
#320	BAY 11-7082	-0.25
#321	Y-27632 2HCl	0.06
#322	TSU-68 (SU6668, Orantinib)	-0.14
#323	Flavopiridol HCl	-0.76
#324	Fingolimod (FTY720) HCl	-0.14
#325	ZSTK474	-0.04
#326	Ralimetinib (LY2228820)	-0.09
#327	TG101209	-0.04
#328	XL388	-0.14
#329	OSI-906 (Linsitinib)	0.37
#330	**AZD7762**	1.04
#331	ZM 336372	-0.14
#332	VE-822	-0.19
#333	GDC-0879	-0.04
#334	PD318088	0.32
#335	GSK1070916	0.32
#336	SSR128129E	0.21
#337	GSK690693	0.01
#338	Acadesine	0.11

#339	Milciclib (PHA-848125)	0.47
#340	AZD2858	-0.35
#341	Alisertib (MLN8237)	0.32
#342	LY2784544	0.47
#343	Dinaciclib (SCH727965)	-0.65
#344	Sorafenib	-0.55
#345	SNS-314 Mesylate	0.72
#346	NVP-BHG712	0.27
#347	Sotrastaurin	-0.70
#348	CEP-32496	0.06
#349	**Regorafenib (BAY 73-4506)**	1.94
#350	Apatinib	0.62
#351	Tyrphostin AG 879	0.42
#352	BMS-345541	0.16
#353	ETP-46464	-0.04
#354	Pacritinib (SB1518)	0.57
#355	P276-00	0.27
#356	Bardoxolone Methyl	0.16

[a]Data are shown as T_m shift (ΔT_m) compared with DMSO control. Compounds which shift T_m more than 1 °C are shown in bold.

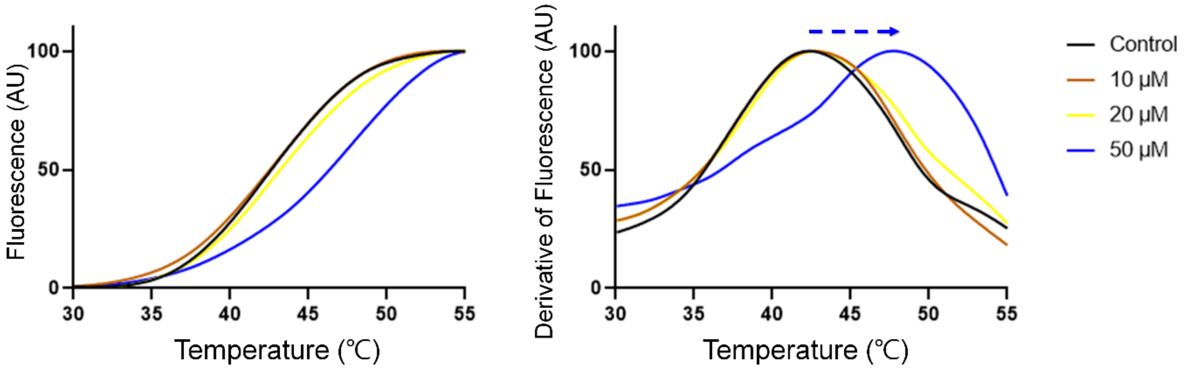

AZD7762	Control	10 μM	20 μM	50 μM
Melting Temperature, T_m (°C)	42.36 ± 0.67	42.65 ± 0.75	42.65 ± 0.61	47.69 ± 0.63
ΔT_m (°C)	-	0.29	0.29	5.23

Figure S1. Thermal shift assay monitoring the interaction between Axl kinase and AZD7762. Left, melting curves, right, corresponding derivative curves. The T_m of Axl in the presence of 2% DMSO (negative control) was ~42.4 °C. While the T_m shifts were <0.5 ºC in the presence of 10 and 20 μM AZD7762, the addition of 50 μM AZD7762 shifted the T_m by ~5.2 °C. AU = arbitrary units. Data are presented as mean ± SD of quadruplicate experiments.

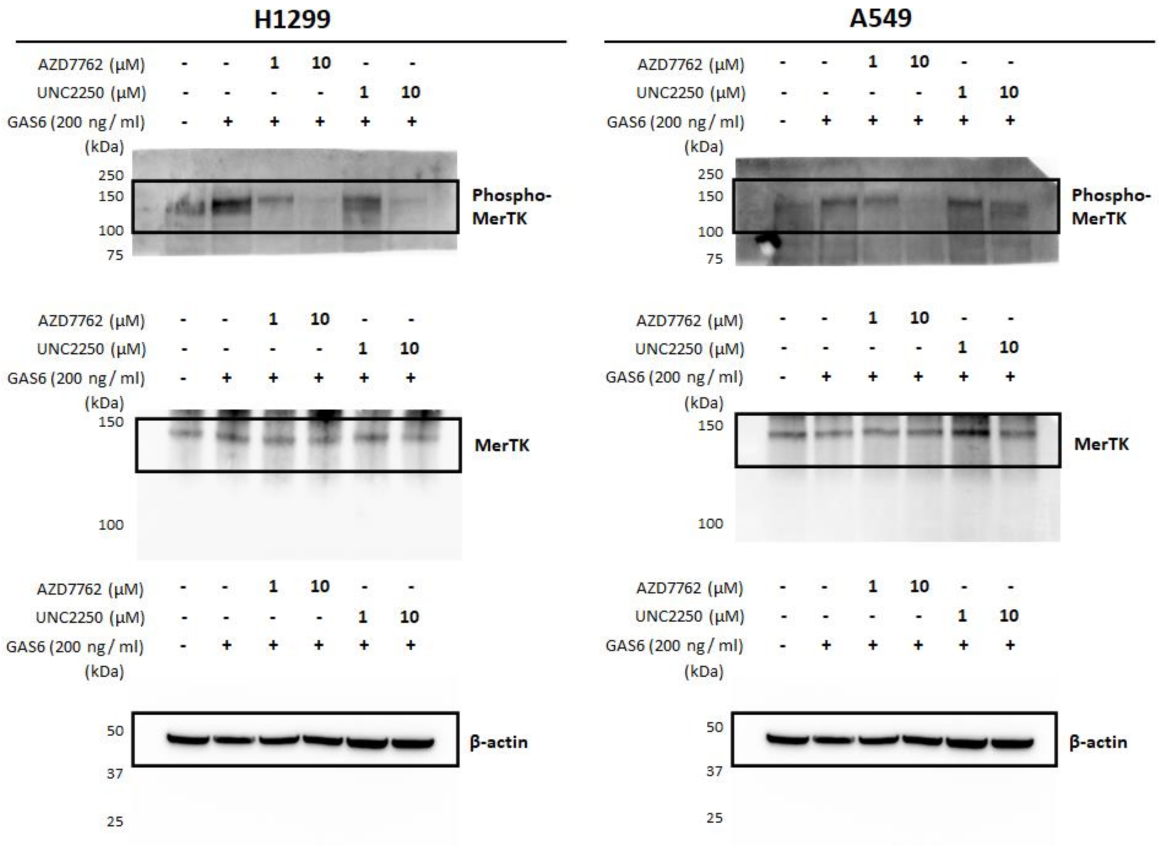

Figure S2. Uncropped images of western blots presented in Figure 3.

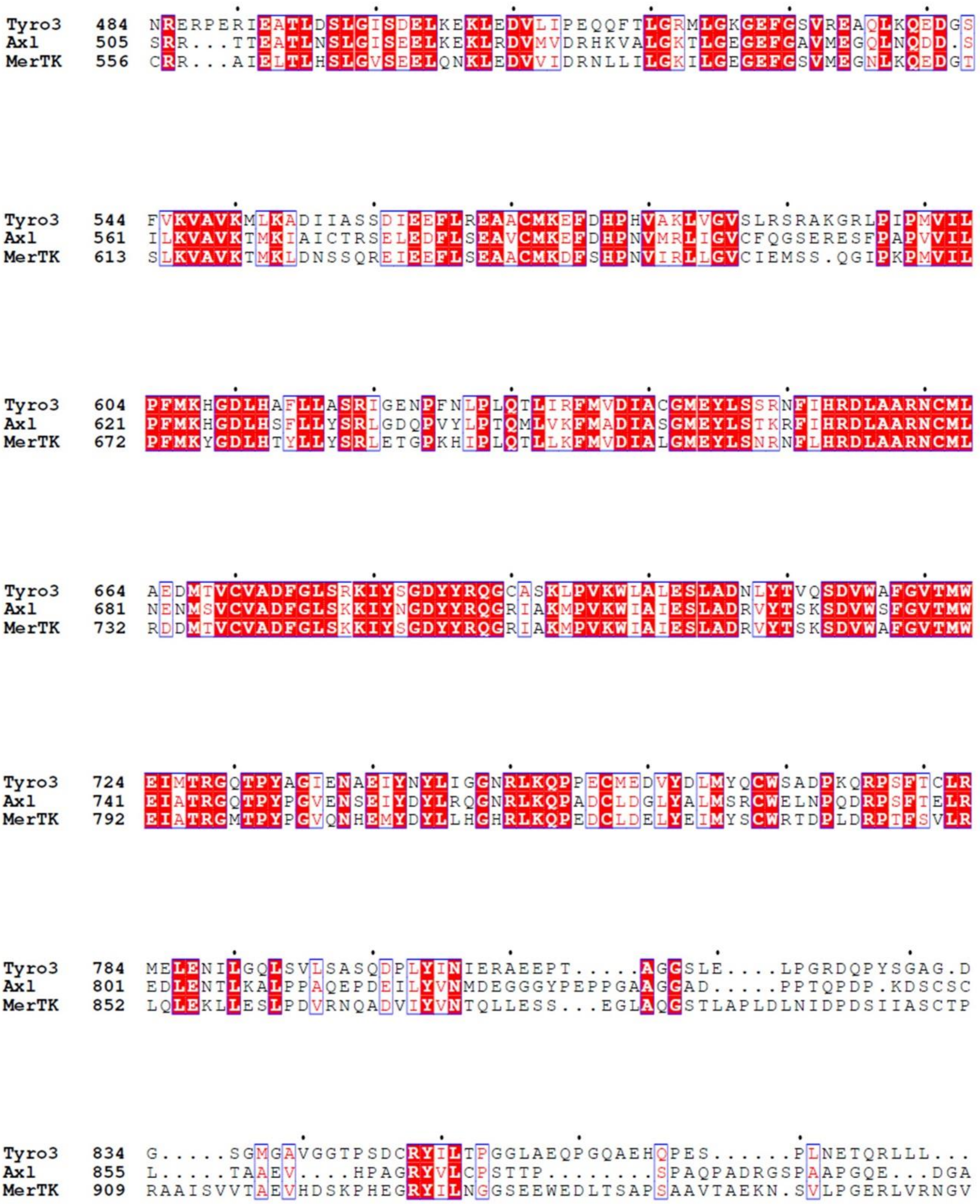

Figure S3. Amino acid sequence alignment of human Tyro3, Axl, and MerTK kinase domains. The residues in blue boxes are strictly (white letters in red background) or highly (red letters) conserved residues.
